# MAPanalyzer: a novel online tool for analyzing microtubule-associated proteins

**DOI:** 10.1093/database/bav108

**Published:** 2015-11-13

**Authors:** Yuan Zhou, Shiping Yang, Tonglin Mao, Ziding Zhang

**Affiliations:** ^1^State Key Laboratory of Agrobiotechnology and; ^2^State Key Laboratory of Plant Physiology and Biochemistry, College of Biological Sciences, China Agricultural University, Beijing 100193, China

## Abstract

The wide functional impacts of microtubules are unleashed and controlled by a battery of microtubule-associated proteins (MAPs). Specialists in the field appreciate the diversity of known MAPs and propel the identifications of novel MAPs. By contrast, there is neither specific database to record known MAPs, nor MAP predictor that can facilitate the discovery of potential MAPs. We here report the establishment of a MAP-centered online analysis tool MAPanalyzer, which consists of a MAP database and a MAP predictor. In the database, a core MAP dataset, which is fully manually curated from the literature, is further enriched by MAP information collected via automated pipeline. The core dataset, on the other hand, enables the building of a novel MAP predictor which combines specialized machine learning classifiers and the BLAST homology searching tool. Benchmarks on the curated testing dataset and the *Arabidopsis thaliana* whole genome dataset have shown that the proposed predictor outperforms not only its own components (i.e. the machine learning classifiers and BLAST), but also another popular homology searching tool, PSI-BLAST. Therefore, MAPanalyzer will serve as a promising computational resource for the investigations of MAPs.

**Database URL:** http://systbio.cau.edu.cn/mappred/.

## Introduction

Microtubule is one of the key components of the eukaryotic cytoskeleton system. *In vivo*, the microtubule is an assembly of multiple protofilaments, and α/β-tubulin heterodimers are adding to or removing from the protofilaments in a dynamic fashion (1–3). The microtubule machinery is not only essential for cell morphogenesis and cell shape maintenance (4, 5), but also plays vital roles in many biological processes, including but not limited to cell division (6), intracellular trafficking (7) and cell signaling (8). In most situations, the collaboration with microtubule-associated proteins (MAPs) is indispensible for microtubules to exert their biological functions (9).

To date, hundreds of MAPs have been discovered, while new types of MAPs keep emerging (10). A few MAPs have been intensively studied, but their working mechanisms and functional implications, in addition to their sequence and structural divergences, appear to be distinct from each other and far from being fully understood. For example, Stathmin has been reported to be able to induce severe depolymerization of microtubules (11). Two models have been proposed to explain its working mechanisms. First, Stathmin can directly sequester free tubulins from polymerizing into microtubules (12). Second, Stathmin binds the growing protofilaments on microtubules with a very strong affinity, keeps it in the bending conformation and prevents it from further assembly (13). Another example is PRC1 from the MAP65 protein family. The MAP65 family is a weakly conserved protein family that bundles microtubules *in vivo* (14, 15). Its representative members in human and *Arabidopsis thaliana* share only about 25% sequence identity. Electronic microscopy images of PRC1, a representative family member from human, indicate that this protein forms an antiparallel dimer through its central rigid domain, providing an explanation for its tendency to bridge two antiparallel microtubules with a restricted gap in-between (16). Nevertheless, PRC1’s *A. thaliana* homologs are likely to induce more divergent forms of microtubule bundles, indicating the mechanism may be more complicated than expected (17). The last example end binding 1 (EB1) protein is representative of an exceptional subset of MAPs, the plus end tracking proteins. A considerable fraction of plus end tracking proteins share a common EB1-binding SxIP motif, highlights the importance of EB1 to organize protein–protein interactions (PPIs) at the microtubule plus end (18). EB1 itself has been demonstrated to bind plus end and promote microtubule growth (19). However, the underlying mechanisms are under long-standing debates. Recently, Maurer *et al.* (20) demonstrate that EB1 decorates and stabilizes microtubule lattice which is enriched for GTP-bound tubulins near the microtubule plus end. Zhang *et al.* (21) further suggest that the decoration of EB proteins on the specific regions of microtubule lattice plays an important role in the microtubule dynamics (21). Therefore, this model provides reasonable interpretation for both the plus-end tracking and the microtubule polymerization promoting activities of EB1.

In contrast to the biological importance and complicated properties of MAPs, computational resources specialized for MAPs (e.g. MAP databases or MAP predictors) are still missing, hampering further experimental investigations. In this study, we attempt to establish a MAP-centered computational analysis tool. We curate a sizable, relatively high confident core dataset by literature reading and construct a MAP predictor based on the representative sequence features extracted from this core dataset. The curated data is further enriched through an automatic annotation pipeline. Finally, the proposed MAP predictor and the collected MAP-related annotations constitute our novel MAP online analysis tool, i.e. MAPanalyzer.

## Results

### The manually curated core dataset

**Figure 1.**
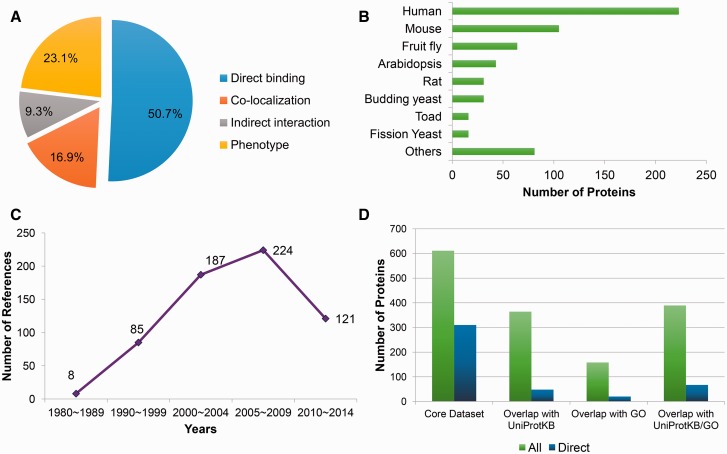
Statistics of the manually curated core dataset. (A) The fraction of different classes of microtubule related proteins; (B) Statistics of source organisms, including human (*Homo sapiens*), mouse (*Mus musculus*), fruit fly (*Drosophila melanogaster*), *Arabidopsis* (*Arabidopsis thaliana*), rat (*Rattus norvegicus*), budding yeast (*Saccharomyces cerevisiae*), toad (*Xenopus laevis*), fission yeast (*Schizosaccharomyces pombe*) and others; (C) Publication year distribution of the supporting references; (D) Overlap with the UniProtKB and Gene Ontology (GO) databases (version of December, 2014), where green bars (‘all’) present the statistics about all of the microtubule-related proteins, while the counts indicted by blue bars (‘direct’) only take proteins that directly bind microtubules into consideration.

Based on literature reading, a dataset of 611 microtubule-related proteins (MRPs) has been collected. This dataset contains four types of MRPs: (i) MAPs, that is, the proteins which directly bind microtubules or tubulins; (ii) Proteins whose gene perturbations induce the alteration of microtubule organization and dynamics *in vivo* (i.e. proteins with microtubule phenotype); (iii) Proteins that colocalize with microtubules and (iv) Proteins indirectly interacting with microtubules, including proteins that interact with a known MAP or presented in the tubulin-containing purification compartment. The MAPs constitute the largest proportion of the core dataset (310 in total; Figure 1A). Among these 310 MAPs, 209 are capable to bind microtubules, 91 bind tubulins, while the remaining 10 interact with EB1 (the core component of microtubule plus end). In terms of experimental evidence, the microtubule cosedimentation assay ranks the top as the standard MAP identification procedure (supporting 54.5% MAPs), followed by popular PPI assays such as coimmunoprecipitation (CoIP), pull down and yeast two hybrid.

The core dataset also features by its species coverage and temporal scope. On the one hand, it records MRPs from 47 species, in which proteins from the generic model organisms unsurprisingly constitute the majority (Figure 1B). But proteins from other organisms like *Bos taurus*, *Chlamydomonas reinhardtii*, *Dictyostelium discoideum*, *Tetrahymena thermophile* and *Trypanosoma brucei* also occupy at least 1% of the data, respectively. The presence of the proteins from non-model organisms is partly related to the fact that some MRPs were firstly identified before the genomic era. Indeed, as shown in Figure 1C, among 625 references supporting the annotations in this dataset, 93 references were published in the last century. As a result, the core dataset provides a more comprehensive collection of MRPs compared with public protein/gene functional annotation databases like UniProtKB (22) or Gene Ontology (GO) (23). As illustrated in Figure 1D, 247 MRPs have not been annotated with keywords ‘tubulin’ or ‘microtubules’ in their function descriptions in UniProtKB (version of December, 2014), 453 MRPs have not been annotated with microtubule-related terms (Supplementary Table S1) in the GO database (version of December, 2014), and together 222 MRPs are not covered by either of them. With respect to the MAPs, only 67 out of 310 MAPs in the core dataset have been annotated as MAPs in either UniProtKB or GO, meaning that 78.4% of the MAPs are exclusively recorded by the core dataset. Therefore, this core dataset would play a fundamental role in constructing the MAP database as well as the MAP predictor in MAPanalyzer.

### Construction of the MAP database

To gather a more comprehensive collection of MRPs, we extended the core dataset by searching the UniProtKB database (22) to find close homologs (sequence identity>50%) of 310 known MAPs in the core dataset. About 2088 homologs were identified, and further classified and annotated according to the experimental evidence (Supplementary Table S1) provided by the GO annotations. There are only six new MAPs among the identified homologs, in line with the above observation that the core dataset already covers many unique MAPs compared with the GO database (Figure 1D). Besides, 37 MRPs that colocalize with microtubules, and 21 MRPs that have microtubule phenotypes have been included, resulting in a substantial growth of MRP entries of the corresponding classes. Finally, most of the homologs do not have experimental evidence for functional associations with microtubules. Such proteins constitute the majority of the extended dataset (Supplementary Figure S1A) and provide a wider organism scope for the dataset (429 genomes in total, Supplementary Figure S1B). These proteins were annotated as the putative MAPs ‘inferred by similarity’, and the microtubule-related electronic GO annotations (if any) were added to our MAP database for better investigations about their potential functions.

As a result, the extended dataset contains 2698 MRP entries supported by 675 references (Supplementary Figure S1C and D). The MRP information can be easily queried from our MAP database (http://systbio.cau.edu.cn/mappred/query.php). The database prefers a UniProtKB or RefSeq accession as the querying keyword, but also keeps compatible with other common types of protein IDs and protein names.

Supplementary Figure S2 shows an exemplary entry in our MAP database, where multiple types of annotations are exhibited, including:

1) Protein IDs and names: When assigning the primary protein accession and protein name, those referred by the authors who first identified the MAPs (or MRPs) are preferred. Therefore, the protein names may be different from those in UniProtKB. Nevertheless, the links to the corresponding accessions in the UniProtKB and RefSeq databases are also provided. Source organism is rarely implied by the protein name. Instead, it is explicitly shown in the database.

2) Function on microtubules: For the MAPs, their literature-reported functions on the microtubule organization and dynamics together with the supporting references are provided.

3) Binding type: As described above (Supplementary Figure S1A), MRPs have been grouped into several classes according to their direct or indirect associations with microtubules. The experimental evidence and supporting references, if any, are also listed.

4) Binding domain and sites: This includes protein domains or segments that influence microtubule binding. Note that this kind of information is usually deduced by the investigations on protein truncating mutations, and therefore does not reach residue-level precision for the most cases.

5) Protein basic information: This includes protein sequence, number of residues, molecular weight and isoelectric point.

6) Domain organization (external information): The domain organization is illustrated according to the Pfam (24) annotations. A summary table of the domains is firstly provided, and users can click the ‘Find it' hyperlink inside the table to find more MRPs containing the same domain. The summary table is followed by a graphical representation of the domain organization in which the domain cartoons are linked to the corresponding Pfam database entry. We also note the emergence of intrinsically disordered proteins in our MRP datasets, and provide a link to the intrinsically disordered region prediction results from the IUPRED server (25).

7) Interaction (external information): Links to the PPI databases BioGRID (26) and IntAct (27) are provided.

8) Reference: The references supporting our manual curation results.

### Establishment and assessment of the MAP predictor

The core dataset also gives rise to the establishment of the MAP predictor. In this section, we will briefly report how the MAP predictor was established and evaluated. Firstly, based on the core dataset, we constructed a nonredundant dataset to train the predictor, which contains 250 positive samples (i.e. known MAPs) and 2500 negative samples (i.e. some randomly selected other proteins involved in the PPIs). The proteins in the training dataset are listed in Supplementary Table S2. We assumed that the MAPs should share some common sequence features, e.g. the sequence motifs. However, few motifs could be retrieved if we directly submitted the sequences of known MAPs to some state-of-the-art motif discovery software tools like MEME (28). Therefore, we devised two specialized motif discovery approaches (see Supplementary Methods for details) and obtained a plethora of representative motifs for MAPs (53 454 in total). We trained a regular support vector machine (SVM) classifier by using these motifs as the input features, and benchmarked the classifier on a non-redundant curated testing dataset including 48 positive samples and 2400 negative samples (Supplementary Table S3). We employed the receiver-operating characteristic (ROC) curve, which plots the sensitivity (true-positive rate) against one minus specificity (false-positive rate), to assess the overall performance of a predictor. The larger area under the ROC curve (AUC) is, the better overall performance a predictor achieves. Generally, the SVM classifier performs well on the curated testing dataset (Supplementary Figure S3, AUC = 0.835). But given the highly imbalanced nature of the curated testing dataset (positive-to-negative ratio = 1:50), the false-positive rate must be properly controlled. That is to say, more attention should be paid to the performance when requiring the specificity ≥90%. With this controlled condition, the performance of the above SVM classifier appears to be not fully satisfactory (Supplementary Figure S3).

One plausible reason is that the extracted 53 454 motifs are somewhat biased. Feature selection methods can be employed to remove the redundant or weak motif features and reduce the bias of the classifier. Three typical feature selection methods have been considered, namely minimum-redundancy maximum-relevancy (mRMR) (29), least absolute shrinkage and selection operator regression (LASSO) (30) and support vector machine recursive feature elimination (SVMRFE) (31). More details about these feature selection methods are available in Supplementary Method. As shown in Supplementary Figure S3, only the features selected by the mRMR method can stably improve the performance when controlling the specificity≥90%. Therefore, we decide to use the mRMR-selected features (463 in total) as our final motif feature set. In addition, to further enhance the robustness of the classifier, the regular SVM was further replaced by a semi-supervised SVM framework, i.e. the Laplacian SVM (lapSVM) (32). One major trait of the lapSVM is the introduction of unlabeled samples, i.e. the samples belong to neither the positive class, nor the negative class. These unlabeled samples are assumed to settle in-between the positive samples and the negative ones, reinforcing the classifying boundary. We noticed that some MRPs in our core dataset have not been reported to bind microtubules, but do have indirect microtubule associations (Figure 1A). These proteins would serve as good candidates for the unlabeled samples. We have gathered a non-redundant set of unlabeled samples from the core dataset of MRPs which colocalize with microtubules, have microtubule phenotypes, or indirectly interact with microtubules (Table S2). As shown in Supplementary Figure S4A, the lapSVM marginally but robustly outperform the regular SVM within the range of specificity≥90%, therefore the lapSVM classifier is finally approved to build our motif-based MAP classifier [i.e. lapSVM(motif)].

In addition to the sharing of representative motifs, MAPs are also likely to have somewhat overall sequence similarity. Since it is difficult for short motif-based features to describe the overall sequence similarity, we employed another sophisticated sequence encoding, the composition of *k*-spaced amino acid pair (CKSAAP) encoding to achieve this goal. As its name implies, the CKSAAP encoding is an extension of simple amino acid pair composition encoding, and considers amino acid pairs with some spaces in-between (e.g. KxK). The CKSAAP has been successfully exploited to accomplish different prediction tasks, including but not limited to the prediction of protein crystallization ability (33), membrane protein type (34) and protein post-translational modification sites (35, 36). Here, we trained a lapSVM classifier based on the CKSAAP encoding [i.e. lapSVM (CKSAAP)]. Similar to the lapSVM(motif), lapSVM(CKSAAP) also exhibits good overall performance on the curated testing dataset (AUC = 0.829), indicating it would be another competitive predictor of MAPs.

In the following paragraphs, we will validate the usefulness of the proposed lapSVM classifiers and describe how these classifiers are finally incorporated into our MAPanalyzer predictor. As mentioned previously, there is no specific MAP predictor available yet, and researchers in the field usually rely on classic homology searching tools like BLAST and PSI-BLAST (37) to predict MAPs. However, whether these generic homology searching tools are competent for predicting novel MAPs have not been comprehensively evaluated. From the curated testing dataset, we exploited BLAST and PSI-BLAST to search the (weak) homologs of the known MAPs presented in the training dataset, and ranked them according to the best hit E-value. The ROC curves can be plotted subsequently by comparing the E-values of positive testing samples and those of negative testing samples. We find that the overall performance of BLAST is not comparable with two lapSVM classifiers (AUC = 0.647 versus 0.833 and 0.829, Supplementary Figure S4A), suggesting BLAST is a conservative method which may not be suitable for identifying new types of MAPs. By exploiting sequence evolutionary profile, the sensitivity of PSI-BLAST is substantially enhanced, with an overall performance nearly equivalent to the lapSVM classifiers (AUC = 0.823). Though neither BLAST nor PSI-BLAST has achieved a better overall performance, it can be found that both of them significantly outperform two lapSVM classifiers when applying very high stringency thresholds (Supplementary Figure S4A). To more precisely compare the predictors, we have applied three stringency thresholds corresponding to the 99, 95 and 90% specificities, respectively. In line with the intuitive observation from the ROC curves, two lapSVM classifiers rank the best at the moderate and high stringency thresholds, with a 4–20% better sensitivity compared with BLAST or PSI-BLAST; but perform the worst at the very high stringency threshold (Table 1). By contrast, BLAST shows very impressive sensitivity at the very high stringency threshold; but does not perform well at the other thresholds (Table 1). These results indicate the potential complementary relationship between BLAST and the lapSVM classifiers. That is, BLAST is superior for finding close homologs, while the lapSVM classifiers are more sensitive to MAPs with weak or insignificant homology. Indeed, after combined with BLAST [i.e. ‘lapSVM(motif)+lapSVM(CKSAAP)+BLAST’], the predictor’s sensitivity at the very high stringency threshold has been considerably improved to 25.0%. Moreover, the combined predictor is not a simple compromise between BLAST and the lapSVM classifiers, but rather significantly outperforms any of its three components at the high and moderate stringency thresholds (Table 1). Finally, the combined predictor also exhibits a better sensitivity than PSI-BLAST at any of the thresholds (Table 1), indicating that the combined predictor should be a promising method to predict novel MAPs.

**Table 1.** Performance comparison on the curated testing dataset at various stringency thresholds

Method	Stringency	Threshold	Sensitivity (%)	Specificity (%)
lapSVM (motif)	Very high	0.42	8.3	99.0
	High	0.1	33.3	95.0
	Moderate	−0.187	45.8	90.0
lapSVM (CKSAAP)	Very high	0.274	6.3	99.0
	High	0.041	37.5	95.0
	Moderate	−0.06	52.0	90.0
BLAST	Very high	30	22.9	99.0
	High	4.22	27.1	95.0
	Moderate	1.54	31.2	90.0
PSIBLAST	Very high	84.4	18.8	99.0
	High	23	29.1	95.0
	Moderate	8.05	39.6	90.0
Combined	Very high	0.121	25.0	99.0
	High	0.019	41.7	95.0
	Moderate	−0.008	56.3	90.0
	Low	−0.042	75.0	80.0

The combined predictor integrates two lapSVM classifiers (based on the representative motifs and the CKSAAP encoding, respectively) with BLAST. For fair comparisons, we have applied three stringency thresholds corresponding to the 99, 95 and 90% specificities of each predictor, respectively. A low stringency threshold is also applied for the combined predictor to enable more sensitive predictions.

Although the above assessments have highlighted the accuracy of the combined predictor, three doubts against these results could be postulated. First, the curated testing dataset is manually collected, and thus may be subjectively biased (e.g. well-studied MAPs may be over-represented). Second, the size of the independent dataset is also limited and may not reflect the *bona fide* accuracy when predicting MAPs from a real genome. Third, the integration with BLAST may not be the optimal choice, since PSI-BLAST would be a better candidate according to its fairly good overall performance.

We addressed all of the above speculations by employing the *Arabidopsis* whole genome dataset. This dataset covers nearly the whole genome of *A. thaliana*, and all of its positive samples were identified from a single proteomics study (38). Therefore, this dataset significantly eliminates the subjective bias, while ensuring the whole genome level coverage. We applied the same threshold values as what were used for the previous independent test. Since the specificities of different predictors become no longer aligned, we employed the Matthews correlation coefficient (MCC) for a comprehensive and fair comparison (Table 2). On the *Arabidopsis* whole genome dataset, the combined predictor exhibits higher overall performance (Supplementary Figure S4B, AUC = 0.727) than BLAST (AUC = 0.562) and PSI-BLAST (AUC = 0.682). When applying certain stringency thresholds, it also significantly outperforms PSI-BLAST and BLAST at nearly all of the thresholds (Table 2). These results suggest the competence of the combined predictor for the genome-wide prediction tasks. Besides, we found the specificities of the combined predictor are much higher than what were estimated from the previous independent testing (Table 1), indicating that the false-positive rate of the combined predictor might be over-estimated, and the previously selected threshold values might be too stringent for the combined predictor. To enable a more sensitive prediction, a low stringency threshold has been added onto the combined predictor (the corresponding performance is also shown in Table 2). Finally, implementation of PSI-BLAST on this dataset also clearly explains why the integration between the lapSVM classifiers and PSI-BLAST was not approved. PSI-BLAST is too time-consuming (Table 2) to be applied for the prediction of MAPs in our web server. In comparison, the lapSVM classifiers and BLAST could finish the prediction on the *Arabidopsis* whole genome dataset in few hours (Table 2).

**Table 2.** Performance comparison on the *Arabidopsis* whole genome dataset at the predefined thresholds

Method	Running time (h)	Stringency	Threshold	Sensitivity (%)	Specificity	MCC
Combined	3	Very high	0.121	9.0	98.8	0.102
		High	0.019	17.7	94.9	0.090
		Moderate	−0.008	28.0	92.0	0.114
		Low	−0.042	48.2	83.9	0.136
BLAST	1.5	Very high	30	8.9	98.5	0.091
		High	4.22	16.6	92.0	0.050
		Moderate	1.54	21.6	88.3	0.049
PSIBLAST	4328	Very high	84.4	9.9	98.8	0.114
		High	23	16.6	92.6	0.055
		Moderate	8.05	26.2	87.9	0.068

The combined predictor integrates two lapSVM classifiers (based on the representative motifs and the CKSAAP encoding, respectively) with BLAST. The thresholds at different stringency levels are as the same as those used in Table 1. The low stringency threshold is also applied for the combined predictor to enable more sensitive predictions. The running time is equivalent to the time consumption under the condition of Dell Power Edge R810 server using a single CPU (Intel Xeon CPU E7-4807, 1.87 GHz).

### Implementation of the MAP server

**Figure 2.**
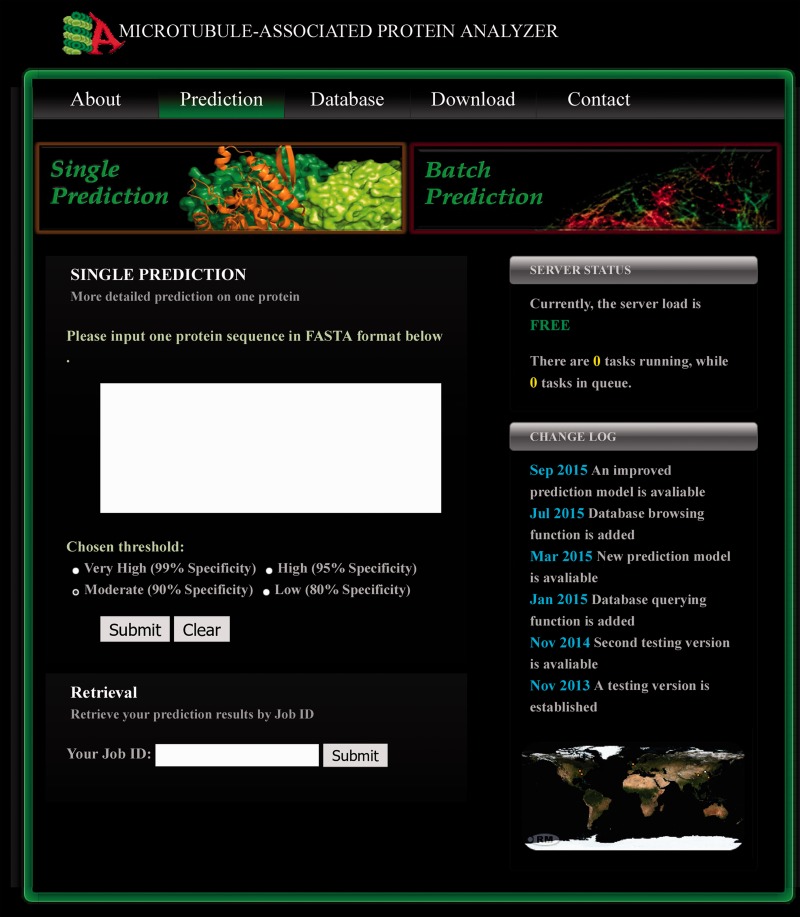
The prediction page of MAPanalyzer. Two prediction modes (i.e. the single prediction mode and the batch prediction mode) are available, and the input form for the former one is shown here. By applying the single prediction mode, a user can submit one protein sequence and the preferred threshold to run prediction. The previous prediction results can be retrieved by inputting the Job ID into the textbox located at the bottom of this prediction page.

Given the accuracy and time-efficiency of the combined predictor, we have made it available in our online server (http://systbio.cau.edu.cn/mappred/index.php). The prediction webpage is shown in Figure 2. As indicated by the navigation buttons, two prediction modes are provided here (i.e. the single prediction mode and the batch prediction mode). By using the default single prediction mode, users can submit one protein sequence in FASTA format and select a preferred stringency threshold. After submission, the prediction task will be immediately carried out, unless the server load is so heavy that the prediction task has to be temporarily appended to the queue. An exemplary prediction result page is shown in Supplementary Figure S5. First, the prediction results and the output scores from two lapSVM classifiers and BLAST are provided. Second, the MRP homologs detected by BLAST (*E* < 10^−^^4^) searching against our database, if any, are also listed, accompanying with the hyperlinks to the corresponding database entries. Third, by comparing the positive samples and negative samples from our curated training and testing datasets, 64 Pfam domains were found to be more frequently appeared in MAPs than randomly selected non-MAPs (Supplementary Table S4). These domains are likely to be associated with microtubule-related biological functions. Therefore, any of these domains found in the query protein are listed in the prediction result page to facilitate further investigations about the functional domains. Finally, among the 53 454 gathered representative motifs for MAPs, 366 motifs were shown to be relatively enriched in the known microtubule binding domains or sites (Supplementary Table S5). Considering aggregation of these motifs may indicate the microtubule binding region, the distributions of these motifs on the query protein sequence are also illustrated in the prediction result page when using the single prediction mode.

Users can also switch to the batch prediction mode by clicking the corresponding navigation button (Figure 2). When using the batch prediction mode, users can upload a protein sequence file and leave an E-mail address where the prediction results will be sent to. No detailed results other than the final prediction scores will be provided in this mode. Finally, users can retrieve their previous prediction results by inputting the job ID into the retrieval form which is located at the bottom of the prediction page (Figure 2).

## Discussion

As demonstrated by recent interactome mapping efforts [e.g. (39, 40)], the cellular interactome is deemed much more complicated than a simple collection of PPIs, and some versatile components from the interactome are highlighted as hub proteins (41). The broad interaction spectrum of a hub protein has intrigued researchers to perform in-depth investigation on some exemplary hub proteins like the calmodulin family proteins (42), the WD domain-containing proteins (43) and the coiled-coil proteins (44). Among them, microtubule (tubulin) should rank as the case of top difficulty. On one hand, the tubulin heterodimer itself is well conserved among the eukaryotic organisms (45). On the other hand, the assembled microtubule is a large protein complex which is renowned for its highly changeable structure (1). The intrinsic dynamic instability of microtubules not only gives rise to the divergence of MAPs, but also sets a barrier against common high-throughput interaction mapping techniques. As a result, knowledge about the MAPs turns out to be scattered amid the studies that focus on individual MAPs. In this study, we established a MAP database by combining extensive manually curation with the automatic annotation pipeline. The resulting MAP database features in its relatively high coverage of known MAP families, in comparison with generic databases like UniProtKB or GO (Figures 1D and Supplementary Figure S2D). Therefore, the complied database would serve as an enriched resource for the systematic studies on MAPs.

What enables a MAP to bind microtubules or tubulins? It is a long-standing question for the biologists in the field. In this study, we assume that MAPs share some representative sequence motifs that can be distinguished from other proteins, and build a MAP predictor based on these motifs accordingly. Despite the accuracy of our final predictor (Tables 1 and 2), the computational framework implies that the determinant for microtubule binding activity seems more complicated than simple motif matching. First, there is no single motif universally applicable for all MAPs. Instead, 53 454 motifs (463 motifs after further selection by the mRMR method) can be derived from the 250 MAPs in the training dataset. Second, a matching of representative motif does not always imply the microtubule binding region, since only 366 out of 53 454 motifs are relatively enriched in the known microtubule binding regions (Supplementary Table S5). On the one hand, these 366 motifs may be related to microtubule binding. Indeed, we note that the positively charged residues are over-represented among the corresponding motif list (Supplementary Table S5), in line with the speculation that conserved positively charged residues are indicators of some microtubule binding regions (46–48). To facilitate the users of MAPanalyzer, the distribution of these 366 motifs on each query sequence is illustrated (Supplementary Figure S5). On the other hand, the vast majority of the representative motifs do not aggregate in the known microtubule binding domains (or regions), indicating some auxiliary sequence motif features may be also helpful for the recognitions of MAPs. Indeed, it is known that for some MAPs, the domain that binds microtubules is distinct from the domain that exerts microtubule function or that interacts with other MAPs (49–51). The sequence features of the latter domains are plausibly captured by the rest of representative motifs. Third and utmost, the success of the ‘lapSVM(motif)+lapSVM(CKSAAP)+BLAST’ combination indicates although the motif information is vital for MAP discrimination, the overall sequence similarity also plays an irreplaceable role. That is to say, the efficiency of the proposed MAP predictor depends both on its exploitation of the motif information and its depiction about the sequence similarity among MAPs through the CKSAAP encoding and BLAST results. Finally, to ensure a wider applicability of our MAP predictor, we here only consider sequence features. Nevertheless, a more sophisticated representation of MAP could be formulated by summarizing the conserved properties among the structures of known MAPs in the near future.

In conclusion, we have reported the establishment of the first specialized computational tool for querying and analyzing MAPs, which contains a sizable MAP database and a novel MAP predictor. Our MAPanalyzer will facilitate and accelerate the related experimental and computational studies on the microtubule system: fundamental but intriguing machinery in the eukaryotic cells.

## Materials and Methods

### Curation of the core dataset

We collected the core dataset of MRPs from the literature. Given the huge amount of available MAP- or MRP-related references (more than 30 000), we followed two compromised approaches to collect MRPs. First, we searched the candidate proteins from the NCBI protein database by using the keyword ‘microtubule associated protein’, and removed the redundant proteins (i.e. >50% sequence identity) by using the BLASTCLUST tool (ftp://ftp.ncbi.nih.gov/blast/documents/blastclust.html). The non-redundant candidate proteins were further manually examined for experimental evidence. Second, we retrieved the abstracts of the MAP- or MRP-related references with the joint keywords of ‘microtubule/tubulin’ and ‘bind/interact/associate’, then curated MRPs by reading the full-text of the references in the filtered list. As described above, MAPs that directly bind microtubules or tubulins, and three other classes of MRPs (i.e. proteins that colocalize with microtubules, indirectly interact with microtubules, and have microtubule phenotype, respectively) were collected. We focused on the curation of experimental evidence, microtubule binding domains (if any) and microtubule functions (if any). Our first round of curation was finished in December, 2012, and the reported latest version of the core dataset was compiled in November, 2014.

### Database construction

The core dataset was further extended by adding the homologs (sequence identity >50%) of known MAPs. The homologs were extracted from the UniProtKB database (22) and annotated according to the experimental evidence (Supplementary Table S1) given by the GO database (version of December, 2014) (23). The extended dataset was loaded as a MySQL database, whose querying interface was constructed and supported by the PHP and Apache techniques.

### Dataset preparation for the MAP predictor

In total, we have prepared one training dataset and two testing datasets for the MAP predictor. In the training and curated testing datasets (Supplementary Tables S2 and S3), the positive samples were the MAPs in the core dataset, while the negative samples were derived from known PPIs. More specifically, in order to gather negative samples, we collected members of the protein complexes recorded in the PDB database (downloaded in September, 2013) (52) and proteins from the species interactome recorded in the BioGRID database (http://www.thebiogrid.org, version 3.2.106) (26). We only considered major source species of the core dataset with a sizeable interactome, including *Homo sapiens*, *Mus musculus*, *Rattus norvegicus*, *Xenopus laevis*, *Drosophila melanogaster*, *Caenorhabditis elegans*, *Arabidopsis thaliana, Saccharomyces cerevisiae* and *Schizosaccharomyces pombe*. The known MRPs indicated by GO annotation (Supplementary Table S1) were excluded from the negative samples. Finally, we applied an intraclass 25% sequence identity cutoff and an interclass 80% sequence identity cutoff to remove redundant sequences. Note that, the interclass 80% sequence identity cutoff implies that a positive sample and a negative sample are allowed to be homologous, and such a relaxed interclass identity cutoff is helpful for rigorous assessment and false-positive control.

By the above procedure, 298 nonredundant positive samples were gathered. 250 of them were randomly selected as the training positive samples, and the rest were included in the curated testing dataset. Subsequently, the nonredundant negative samples were randomly added to either of the datasets, until the 1:10 and 1:50 positive-to-negative ratios were reached for the training and curated testing datasets, respectively. Finally, other MRPs in the core dataset constituted the unlabeled samples in the training dataset, which is only used by the lapSVM classifiers. The redundant sequences among the unlabeled samples were also removed by using BLASTCLUST with an intra-class 25% sequence identity cutoff.

We also employed the *Arabidopsis* whole genome dataset as a more comprehensive testing dataset. The positive samples in this dataset were the potential MAPs identified by one proteomic assay (38), and other proteins from the *A. thaliana* genome constituted the negative samples. Proteins presented in the training dataset were removed from this testing dataset. Note that for a realistic evaluation of the genome-wide prediction performance, no redundancy removal procedure was applied to this dataset.

### Predictor establishment and assessment

For the motif-based classifiers, we extracted and selected representative motifs as the input features of the regular SVM and the lapSVM classifiers (detailed procedure is available in the Supplementary Method). The representative motifs were encoded in the binary fashion, that is, if the protein is matched by one motif, the corresponding feature value is 1, otherwise 0. The regular SVM was established by using the LIBSVM software with the radius basis function kernel (53), while the lapSVM training and predicting were implemented by translating the source code provided by the original authors (32) into R scripts (because the R script is suitable for the implementation in our online server). The parameters of these machine learning classifiers were optimized through 5-fold cross-validations, similar to our previous study (54). The optimized parameters are listed in Supplementary Table S6.

For the CKSAAP-based lapSVM classifier, we first encode the protein sequence according to the CKSAAP encoding scheme. Briefly speaking, the CKSAAP encoding describes protein sequence using the composition of *k*-spaced amino acid pairs. The integer *k* is the number of spaces between an amino acid pair, ranging from 0 to *k*_max_. In this study, *k*_max_ was optimized as 1, i.e. only amino acid pairs with 0 or 1 space in-between were counted. Detailed calculation procedures of the CKSAAP encoding have been explicitly described in previous studies (33, 34). The CKSAAP-based lapSVM classifier was trained in the same way as the motif-based lapSVM classifier. The optimized parameters for the CKSAAP-based lapSVM are also listed in Table S6.

For BLAST and PSI-BLAST, we searched the database of training positive samples by using the testing sequence (or its sequence profile when running PSI-BLAST). The sequence profile is generated by running PSI-BLAST *in priori* against the NCBI nr90 database with the common parameters ‘-h 0.001 -j 2’. For each query, the best hit E-value (*Ev*) was extracted from the output file of the BLAST or PSI-BLAST program, and further transformed into *pEv* as:

(1) $$pEv=\{\begin{array}{lll} 200 & \text{if }Ev\leq 10^{-200} & \\ -\log_{10}Ev & {\text{if 10}}^{-200}<Ev<10^{3} & \\ -3 & \text{if }Ev\geq 10^{3}\text{ or no hit found} & \end{array}$$

As described in the Results, the combination of two lapSVM classifiers (which are based on representative motifs and CKSAAP encoding, respectively) and BLAST results in a more powerful predictor. The combination of these three predictors was achieved by the weighted averaging of the normalized decision score of the motif-based lapSVM (*Ds*_Motif_), the normalized decision score of the CKSAAP-based lapSVM (*Ds*_CKSAAP_) and the transformed BLAST E-value (*pEv*), defined as

(2) $$\begin{array}{l} S_{combined}=\frac{1}{3}(\alpha_{Motif}\cdot \frac{1-e^{-Ds_{Motif}}}{1+e^{-Ds_{Motif}}}+\alpha_{CKSAAP}\cdot \frac{1-e^{-Ds_{CKSAAP}}}{1+e^{-Ds_{CKSAAP}}}\\ +\alpha_{BLAST}\cdot \frac{pEv}{10}) \end{array}$$

where *S*_combined_ is the final output score of the combined predictor, and *α*_Motif,_ *α*_CKSAAP_ and *α*_BLAST_ are the weights for the corresponding score terms. *α*_Motif,_ *α*_CKSAAP_ and *α*_BLAST_ have been preliminarily optimized as 0.35, 0.5 and 0.15, respectively.

As previously mentioned, the established predictors were benchmarked on the two independent testing datasets. We measured the sensitivity, specificity and MCC at certain stringency thresholds. These performance indicators could be calculated as,

(3) $$Sensitivity=\frac{\text{TP}}{\text{TP}+\text{FN}}$$

(4) $$Specificity=\frac{\text{TN}}{\text{TN}+\text{FP}}$$

(5) $$\text{MCC}=\frac{\text{TP}\times \text{TN}-\text{FP}\times \text{FN}}{\sqrt{\left(\text{TP}+\text{FP})\times (\text{TP}+\text{FN})\times (\text{TN}+\text{FP})\times (\text{TN}+\text{FN}\right)}}$$

where TP, FP, TN, FN stand for the count of true positive, false positive, true negative, false negative, respectively. We also employed the ROC curve (55) to measure the overall performance of different predictors. The ROC curve plots sensitivity (true-positive rate) against one minus specificity (false-positive rate), as the classification threshold varies. The AUC is used to evaluate the overall performance of a predictor.

## Supplementary Material

Supplementary Data
