# Supplementary material for: MAPanalyzer: a novel online tool for analyzing microtubule-associated proteins
Source: Database (Oxford). 2015 Nov 13;2015:bav108. doi: 10.1093/database/bav108 (PMC4644220; doi:10.1093/database/bav108)
Supplement: Supplementary Data [file supp_2015_bav108_index.html]

MAPanalyzer: a novel online tool for analyzing microtubule-associated proteins — Supplementary Data 

# MAPanalyzer: a novel online tool for analyzing microtubule-associated proteins

## Supplementary Data

files

- Supplementary Data - doc file
- Supplementary Data - doc file
- Supplementary Data - xls file
