# Supplementary material for: MAPanalyzer: a novel online tool for analyzing microtubule-associated proteins
Source: Database (Oxford). 2015 Nov 13;2015:bav108. doi: 10.1093/database/bav108 (PMC4644220; doi:10.1093/database/bav108)
Supplement: Supplementary Data [file supp_bav108_Supplementary_Figures_database_rv2.doc]

**Supplementary Figures**

**
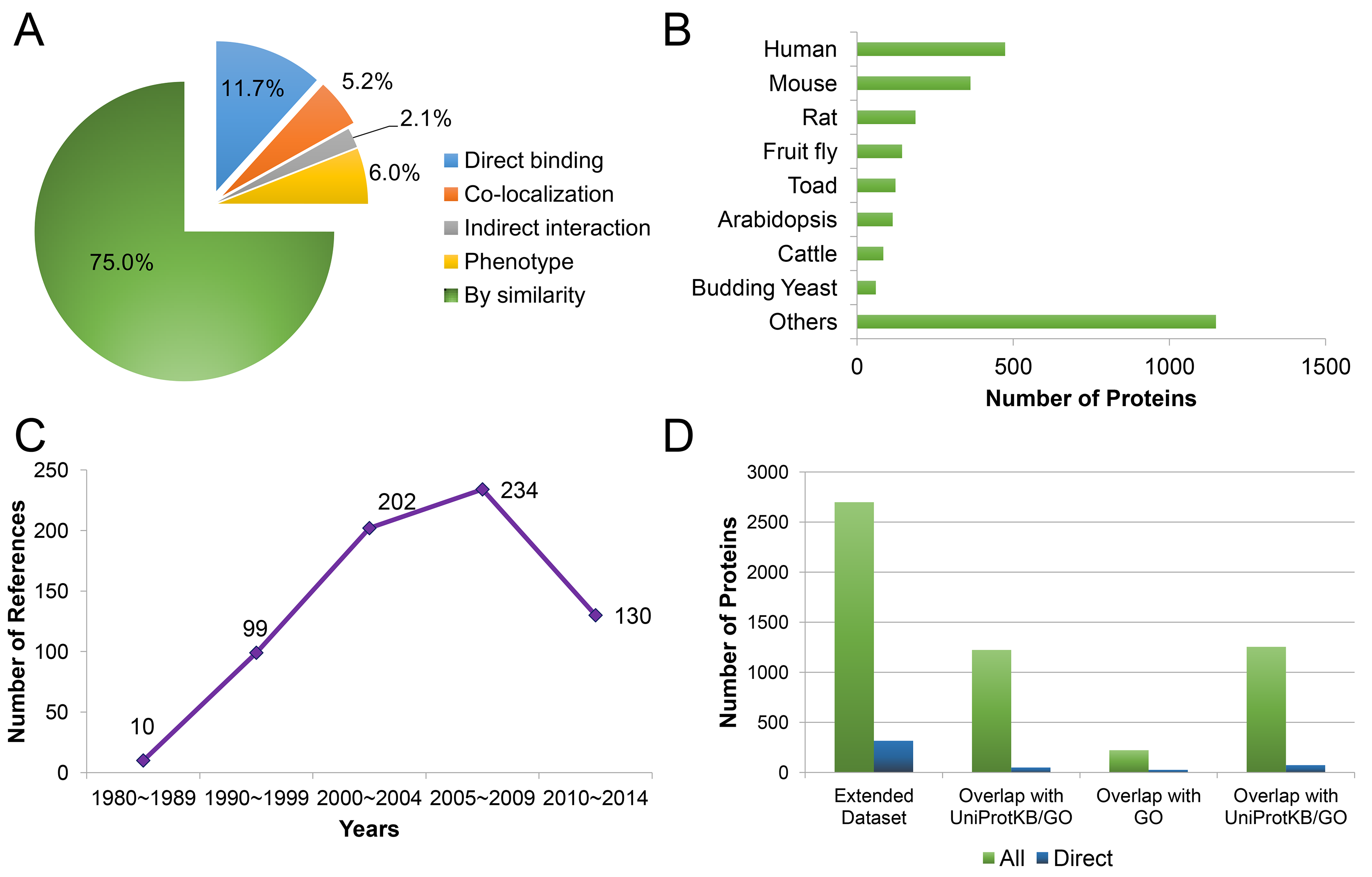
**

**Figure S1.** Statistics of the extended dataset.

(A) The fraction of different classes of microtubule related protein; (B) Statistics of source organisms, including human (*Homo sapiens*), mouse (*Mus musculus*), rat (*Rattus norvegicus*), fruit fly (*Drosophila melanogaster*), toad (*Xenopus laevis*), Arabidopsis (*Arabidopsis thaliana*), cattle (*Bos taurus*), budding yeast (*Saccharomyces cerevisiae*) and others; (C) Publication year distribution of the supporting references; (D) Overlap with the UniProt and Gene Ontology (GO) databases (version of December, 2014), where green bars ("all") present the statistics about all of the microtubule related proteins, while the counts indicted by blue bars ("direct") only take proteins that directly bind microtubules into consideration.

**
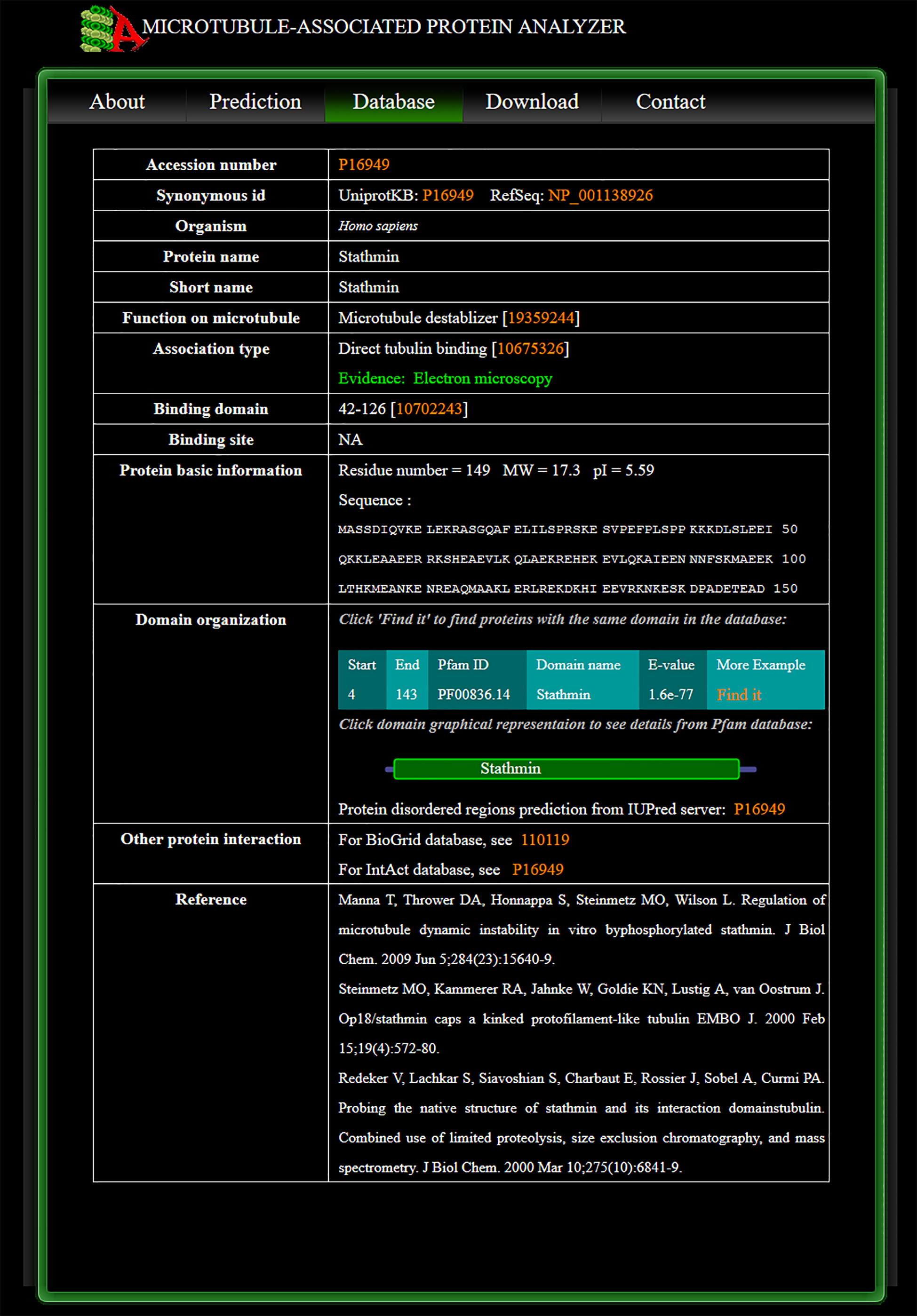
**

**Figure S2.** A sample database entry page in MAPanalyzer.

**
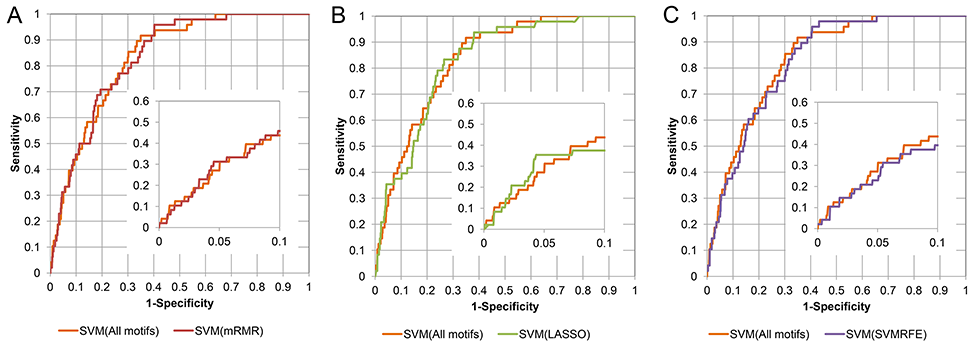
**

**Figure S3.** ROC curves for the comparison of performance on the curated testing dataset. The models trained with the selected motifs and that trained with all motifs are compared in the following order: (A) mRMR-selected motifs *vs.* all motifs; (B) LASSO-selected motifs *vs.* all motifs; (C) SVMRFE-selected motifs *vs.* all motifs.

**
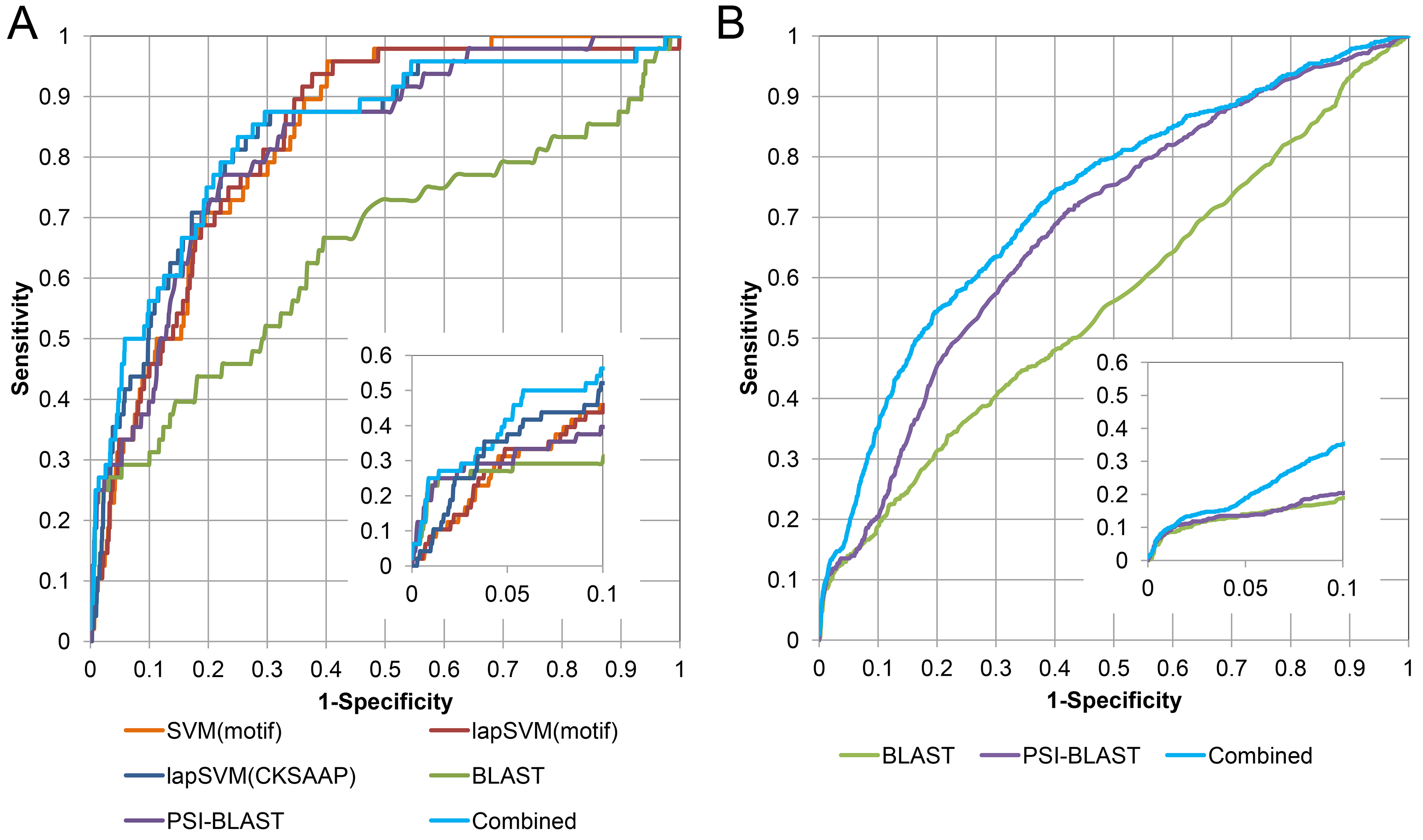
**

**Figure S4.** ROC curves illustrating the performance of different predictors on the testing datasets. (A) ROC curves based on the benchmarking results on the curated testing dataset. (B) ROC curves based on the benchmarking results on the *Arabidopsis* whole genome dataset. The combined predictor is the combination of the motif-based lapSVM classifier, the CKSAAP-based lapSVM classifier and BLAST [i.e. “lapSVM(motif) + lapSVM(CKSAAP) + BLAST”]. Note that, due to the unbalanced positive-to-negative ratio of the testing datasets (1:50 and 1:37, respectively), the performance when requiring specificity≥90% are emphasized and shown at the right-bottom corner of the plots.


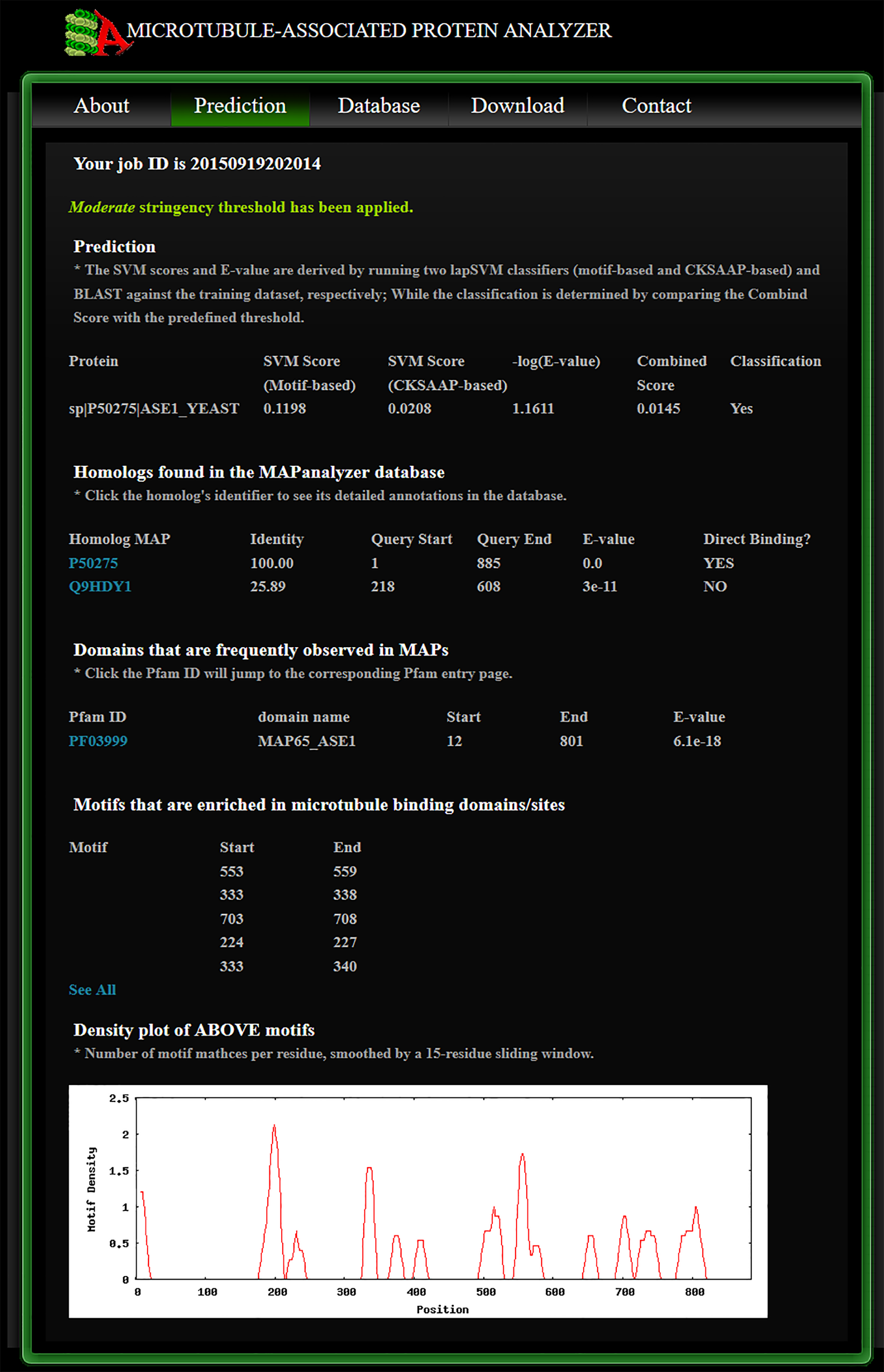


**Figure S5.** An exemplary result page by using the single prediction mode of MAPanalyzer.
