# Supplementary material for: MAPanalyzer: a novel online tool for analyzing microtubule-associated proteins
Source: Database (Oxford). 2015 Nov 13;2015:bav108. doi: 10.1093/database/bav108 (PMC4644220; doi:10.1093/database/bav108)
Supplement: Supplementary Data [file supp_bav108_Supplementary_Methods_database_v2.doc]

**Supplementary Methods**

**Motif extraction approaches**

We followed two complementary approaches to extract representative motifs of MAPs. In the first approach, we extracted representative motifs from the MAP sequences in the training dataset and their close homologs. For each positive sample (MAP) in the training dataset *m_i_*, we gathered its homologs in NCBI nr90 database by using BLAST with the 50% identity cutoff. Redundant sequences in the BLAST results were further removed by BLASCLUST with the 50% sequence identity cutoff. As a result, for each *m_i_*, we obtained its homolog protein set ***H_i_***.

The representative motif of *m_i_* can be extracted from ***M***, a set of proteins which contains ***H_i_*** and other related homolog protein sets which are iteratively added during the progress of the algorithm:

Step 1: For each *m_i_* belongs to the ***Tp*** (i.e. the set of positive samples), let ***M***=***H_i_***.

Step 2: Find the sequence most similar to *m_i_* in ***Tp***, denote it as *m_j_* (*m_j_* ≠ *m_i_*). The similarity between two positive samples is measured by the Euclidean distance between their amino acid frequency vectors.

Step 3: Append the homolog protein set of *m_j_* (i.e. ***H_j_***) to ***M***.

Step 4: Extract 10 motifs of *w* amino acid length from ***M*** by using MEME software , setting the running mode by the “-oops” argument (i.e. one occurrence per sequence). The MEME software assigns an E-value to each motif, and the minimum E-value among the 10 motifs is found out and denoted as *minEv*.

Step 5: If current *minEv* is lower than the previous one, remove *m_j_* from ***Tp*** and repeat Steps 2~4. Otherwise, the algorithm is converged, then the previously derived ten motifs are reported as the optimized output motifs (this condition also implies that at least two iterations should be performed before the convergence of the algorithm).

Five runs of the algorithms were performed independently with the motif length *w* set as {5,6,7,8,9}, respectively. We used the exact length rather than a length range as the input parameter for MEME, because we found MEME strongly favored longer motifs and ignored shorter ones when a length range was applied. Besides, we also did not consider motifs shorter than 5 in this approach, as we found that for short motifs, the above algorithm often failed to converge in acceptable time. Nevertheless, short motifs could be gathered by another approaches described below.

In the second approach, we exhaustively searched motifs overrepresented in the positive samples as:

Step 1: Exhaustively generate motifs of 9 amino acids with 3 fixed characters and 6 wildcard characters. The fixed characters match exact amino acids, while the wild card characters match any amino acid.

Step 2: For each motif, search against the training dataset. If it matches no less than 3 positive samples and no more than 250 negative samples (i.e. 10% of the total negative samples in the training dataset), the motif will be retained. Otherwise, the motif will be discarded.

Step 3: One retained motif *υ* may contain *x* wildcard characters {*v_1_*, *v_2_*, …, *v_x_*}. Each wildcard character *v* can be further specified by substituting it with an exact amino acid *u*, resulting in a new motif (i.e. *motif_v,u_*). Whether the substitution is approved is determined by the enrichment score calculated as:

$ES_{u}=\sum_{m_{i}\in Tp} \left[1\cdot\delta\left(motif_{v,u},m_{i}\right)+\frac{1}{\tau_{i}}\cdot\sum_{o\in H_{i}} \delta\left(motif_{v,u},o\right)\right]-0.2\cdot\sum_{n_{i}\in Tn} \delta\left(motif_{v,u},n\right)$ (S1)

where ***Tp*** and ***Tn*** are the positive and negative sample sets, respectively. For each *m_i_* that belongs to ***Tp***, its homolog protein set is denoted as ***H_i_***. For any protein *a*, the term *δ*(*motif_v,u_*, *a*) equals to 1 if the motif matches this protein, equals to 0 otherwise. To avoid bias, recurring motifs in one protein sequence are counted only once. Therefore, the equation S1 turns out be a weighted summing of the counts of the proteins matched by the *motif_v,u_* in different sets. The weight for a positive sample *m_i_* is set to 1, and the weight for its homologs is set as 1/*τ_i_*, where *τ_i_* is the amount of its homologs. That is to say, if the *motif_v,u_* appears in one positive sample and all of its homologs, the enrichment score *ES_u_* will gain 2 points. Considering the 1:10 positive-to-negative ratio of the training dataset, the weight for a negative sample *n_i_* is naturally set to -0.2 to cancel the score gaining from motif matches in a positive sample and its homologs. A substitution is approved if its *ES_u_* >0, and all of the approved substitution were summarized to generate a specified motif (e.g., as A[DEST]KK[QN]K). However, when no substitution is approved, or more than five substitutions are approved, this wildcard is deemed difficult to be further specified，and remains unchanged (e.g., as AxKKxK).

Step 4: All remaining wildcard characters in the end of the motifs were truncated, to enable motifs whose length span 3 to 9 amino acid residues.

Step 5: Since the retained motifs after Step 4 were still of huge amount (more than 0.3 million), a preliminary filtering against redundant motifs is necessary. Two motifs *υ_1_* and *υ_2_* are considered redundant if their matched positive samples considerably overlap, as measured by the Jaccard index (*JI*):

$JI=\frac{\left|Tp(\upsilon_{1})\bigcup Tp(\upsilon_{2})\right|}{\left|Tp(\upsilon_{1})\bigcap Tp(\upsilon_{2})\right|}$ (S2)

where ***Tp***(*υ_1_*) and ***Tp***(*υ_1_*) are the subsets of positive samples matched by *υ_1_* and *υ_2_*, respectively. If *JI*>0.25, two motifs were considered redundant. This *JI* threshold was preliminarily optimized in order to achieve a balance between the retained motif information and the computational burden.

If *υ_1_* and *υ_2_* are redundant, we discarded *υ_2_* if it meets either of the two following conditions:

a) |***Tp***(*υ_1_*)| > |***Tp***(*υ_2_*)| AND |***Tn***(*υ_2_*)| - |***Tn***(*υ_1_*)|≥5, where ***Tn***(*υ_1_*) and ***Tn***(*υ_2_*) are the subsets of negative samples matched by *υ_1_* and *υ_2_*, respectively. This condition assures the motif *υ_1_* is relatively overrepresented in the positive samples compared with *υ_2_* .

b) |***Tp***(*υ_1_*)| = |***Tp***(*υ_2_*)| AND |***Tn***(*υ_2_*)| - |***Tn***(*υ_1_*)|<5 AND *WD*(*υ_1_*) > *WD*(*υ_2_*), where the WDs are the wobbling degrees. For each wildcard character, the motif gain 1 points of *WD*, e.g. *WD*(AxKKxK)=2. For each specified wobbling character with *n* possibilities, the motif gain a WD scored in the affine fashion as 0.5+0.1*(n-1), e.g. *WD*(A[DEST]KK[QN]K)=(0.5+0.1*3)+(0.5+0.1*1)=0.8+0.6=1.4. This condition implies that the *υ_1_* is not more overrepresented in the positive samples compared with *υ_2_*, but its highly wobbling composition may enable more sensitive motif matching.

**Feature selection procedures**

The above motif extraction procedures resulted in 53454 representative motif features of MAPs that needed further selection to remove weak, biased and redundant features. We have tried three popular feature selection methods and chose the one achieving the best independent testing performance, when the specificity≥90% is required. These feature selection methods are briefly introduced as follows:

a) Minimum-redundancy maximum-relevancy (mRMR). This method was proposed by Ding and Peng . Its optimization objective for the feature set ***F*** is clearly demonstrated by the name of the method, i.e. maximizing the difference between total relativity (*REL*) and total redundancy (*RED*):

(S3)

where MI indicates mutual information, ***y*** is the class label vector, while ***x(f_i_)*** and ***x(f_j_)*** are the feature value vectors for features *f_i_* and *f_j_*, respectively. We used the downloadable software provided by the original authors to implement the mRMR method. This software outputs the top 500 features only, and we applied a feature score threshold of 0.2 to ensure most of the top 500 feature are included in the final set of the selected features.

b) Least absolute shrinkage and selection operator regression (LASSO). Distinct from regular regression techniques, the LASSO introduces a specific penalty term against the amount of the variables (features) as:

(S4)

The latter term in the equation S4 implies variables (features) with non-zero weight *β* should be avoided. That is to say, redundant variables (features) and those unrelated to class label will be assigned as *β=0*, and every variable (feature) with non-zero weight should be selected. We exploited the glmnet package in R to run LASSO . For more reliable results, we used three-fold cross-validation to train the LASSO model. The three-fold cross-validation procedure was repeated 100 times, and the largest set of the selected feature in the 100 times of running was finally chosen.

c) Support vector machine recursive feature elimination (SVMRFE). Briefly, this method recursively eliminates weak features from the feature set ***F***, in order to achieve the best performance of the SVM trained with the selected set of features . Before descriptions of the detailed algorithm, two key parameters should be clarified. The first one is the importance score of a feature (i.e. *w*^2^), which is calculated as

(S5)

where *y_i_*, *α_i_* and *x_i_* are the class label, weight and feature value of the *i*-th sample, respectively. Because the *w* can be either positive or negative, the square operation in equation S5 ensures a comparable importance score. Note also that we trained SVM with the *linear* kernel when running SVMRFE, since only for the SVM trained with the *linear* kernel, the calculation of the importance score *w*^2^ as shown in the equation S5 is valid.

In addition, the performance of the trained SVM can be evaluated by the balanced accuracy (*BAC*),

(S6)

where *TP*, *FP*, *TN*, *FN* stand for the counts of true positive, false positive, true negative, false negative, respectively.

Finally, considering the large amount of our motif features (53454 in total), a modified version of SVMRFE was performed as:

Step 1: Train a SVM model with feature set ***F*** (***F*** contains all of the feature in the first round of iteration), estimate its *BAC* through three-fold cross-validation.

Step 2: Since three SVM models can be generated during a three-fold cross-validation, three importance scores (*w*^2^) are derived for each feature. The features are sorted by their average importance scores in descent order.

Step 3: Eliminate the worst *x* features from ***F***. *x* equals to 5% of the amount of the remained features when number of the remained features |***F***| ≥1000, but equals to 1 when |***F***|<1000. This configuration is helpful to accelerate the algorithm progression toward the optimal solution, but to emphasize accuracy when the optimal solution is nearly achieved.

Step 4: Stop iteration if |***F***|<100, otherwise repeat Steps 1~3.

Step 5: Find the ***F_best_*** that achieves the highest BAC during the iterations as the final set of selected features.

**References for Supplemental Methods**

1. Bailey, T.L., Boden, M., Buske, F.A.*, et al.* (2009) MEME SUITE: tools for motif discovery and searching. *Nucleic Acids Res.*, **37**, W202-208.

2. Ding, C., Peng, H. (2005) Minimum redundancy feature selection from microarray gene expression data. *J. Bioinform. Comput. Biol.*, **3**, 185-205.

3. Friedman, J., Hastie, T., Tibshirani, R. (2010) Regularization paths for generalized linear models via coordinate descent. *J. Stat. Softw.*, **33**, 1-22.

4. Guyon, I., Weston, J., Barnhill, S.*, et al.* (2002) Gene selection for cancer classification using support vector machines. *Mach. Learn.*, **46**, 389-422.
